# Effect of body mass index on surgical times of lumbar laminoplasty and lower limb arthroplasties

**DOI:** 10.1186/s12891-019-2788-5

**Published:** 2019-09-06

**Authors:** Kengo Harato, Mitsuru Yagi, Nobuyuki Fujita, Shu Kobayashi, Akihito Ohya, Kazuya Kaneda, Yu Iwama, Masaya Nakamura, Morio Matsumoto

**Affiliations:** 1 Department of Orthopedic Surgery, Keio University School of Medicine, 35 Shinanomachi, Shinjuku-ku, Tokyo, 160-8582 Japan; 2 Keio Orthopedic Advancing Squad for the Interactive Study (Keio OASIS), Tokyo, Japan

**Keywords:** Degenerative disease, Obesity, Lumbar canal stenosis, Osteoarthritis, Operation time

## Abstract

**Background:**

Obesity is an important factor affecting incidence and development of musculoskeletal degenerative changes. In addition, obese patients are considered less favorable surgical candidates for decompression surgery in degenerative lumbar spinal canal stenosis and lower limb arthroplasty. The purpose was to assess disease characteristics of lumbar spinal canal stenosis as well as lower limb osteoarthritis, and to investigate surgical times based on body mass index (BMI) in lumbar decompressive surgery and lower limb arthroplasties.

**Methods:**

A total of 1161 patients with a diagnosis of lumbar canal stenosis (LCS), hip osteoarthritis (HOA) and knee osteoarthritis (KOA) were enrolled. The present investigation was conducted as a retrospective study using routinely collected data. All patients underwent primary decompressive surgery (laminoplasty: LAM) or lower limb arthroplasty (total hip arthroplasty: THA and total knee arthroplasty: TKA). All of the patients were divided into 3 groups based on BMI (kg/m^2^) (Group A: ≤ 24.9; Group B: 25–29.9; Group C: ≥ 30) within each disease category. To assess disease characteristics, age, gender, and BMI were evaluated for each disease category. Moreover, surgical times for LAM, THA and TKA were also assessed based on BMI classification.

**Results:**

A total of 269, 470, and 422 patients were allocated to the HOA category, the KOA category, and the LCS category, respectively. The KOA category included the oldest patients and largest BMI, compared to the HOA and the LCS categories. Regarding gender difference, LCS was more common in males than in females, while opposite phenomenon was observed in the HOA and the KOA categories. The heaviest group (Group C) was significantly younger than Groups A or B in TKA and LAM. Surgical time was significantly longer in patients with overweight or obese patients than in those with normal weight in TKA and LAM, while BMI didn’t affect the time in THA.

**Conclusions:**

Disease characteristics of the KOA category and the LCS category were notably affected by BMI, and surgical times in TKA and LAM were significantly longer for overweight or obese patients, whereas THA was less affected by BMI concerning disease characteristics and surgical time.

## Introduction

Generally, obesity affects one-third of the adult U.S. population and is associated with numerous clinical problems [1–3]. The impact of obesity on musculoskeletal problems, including degenerative spinal and joint diseases at wight-bearing joints, has been well documented so far [2–14]. Obesity is a modifiable risk factor regarding incidence and development of musculoskeletal degenerative changes. For instance, Reyes C et al. conducted a population-based cohort study using 1,764,061 subjects and concluded that being overweight or obese could increase the risk of hand, hip, and knee OA, with the greatest risk in the knee [13]. On the other hand, Reijman M et al. perfomed a similar population-based cohort study using 3585 people aged > 55 years, and indicated that body mass index (BMI) was associated with the incidence and progression of knee osteoarthritis, while BMI was not associated with the incidence and progression of hip osteoarthritis [12]. Therefore, increased BMI is strongly correlated with both knee osteoarthritis (KOA) and hand osteoarthritis, whereas several studies have found no association between BMI and hip osteoarthritis (HOA) [12]. In addition, obese patients are considered less favorable surgical candidates for decompression surgery in degenerative lumbar spinal canal stenosis (LCS) and lower limb arthroplasty including hip and knee joint [2, 15–17]. Some reasons include increased surgical time, longer length of hospital stay and more postoperative complications for obese patients, compared to patients with normal weight. However, little attention has been paid to surgical times of decompression surgery in degenerative lumbar spinal canal stenosis and total joint arthroplasties in lower limb osteoarthritis based on BMI.

The purpose of the present study was to assess disease characteristics of lumbar spinal canal stenosis as well as lower limb osteoarthritis, and to investigate surgical times based on BMI in lumbar decompressive surgery and lower limb arthroplasty. It was hypothesized that disease charactersitics and surgical times would be strongly affected by BMI for each category.

## Methods

### Participants

**Fig. 1:**
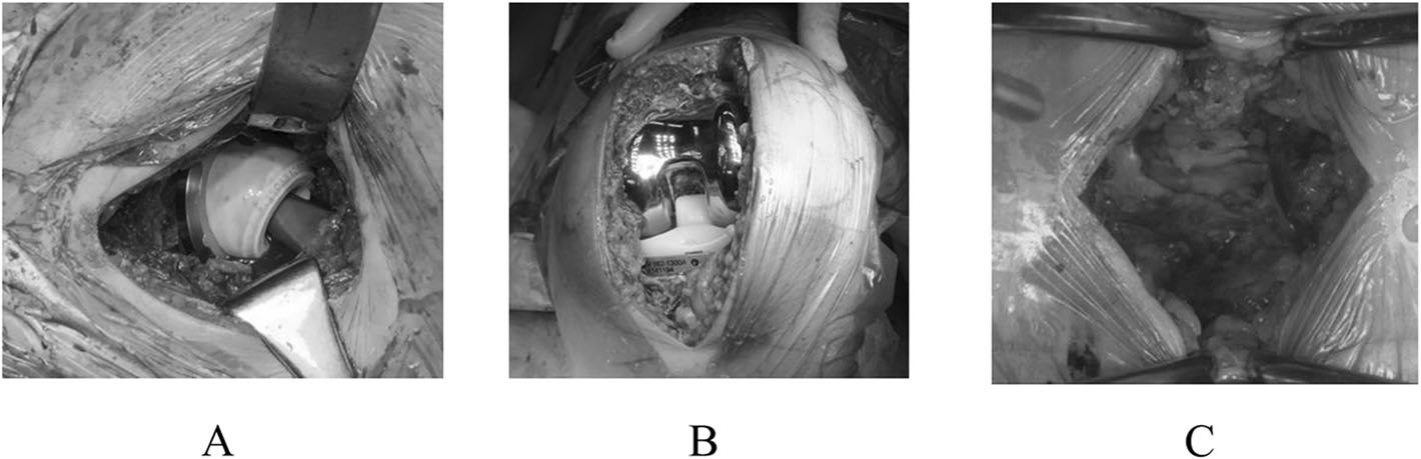
Each surgery. **a**. THA, **b**. TKA, **c**. LAM

A total of 1161 patients with a diagnosis of LCS, HOA and KOA were enrolled in the present study. Data collection was done from 2014 to 2018. The present investigation was conducted as a retrospective study using routinely collected data. Diagnosis was done by senior orthopaedic surgeons based on physical examination, plain radiograph, Computed Tomography and Magnetic Resonance Imaging if necessary. All patients underwent primary decompressive surgery (laminoplasty: LAM) or lower limb arthroplasty (total hip arthroplasty: THA and total knee arthroplasty: TKA) (Fig. 1). Each category had different patient group. Patients with rheumatoid arthritis were excluded from the analysis. Revision surgery and primary surgery with additional procedure such as femoral/tibial osteotomy or repair of dural membrane during the surgery were also excluded. All the surgeries were done at the high-volume institution by middle- or high-volume surgeons, including 6 surgeons for LAM, 3 surgeons for THA, and 3 surgeons for TKA. Surgical approaches in total knee and hip arthroplasty were standard parapatellar and anterior approach, respectively. All TKAs and THAs were done without special devices such as navigation or patient-matched instrument. Similarly, well-trained spine surgeons performed single level LAM using posterior approach in supine position. All methods and procedures were approved by our institution’s ethics committee.

### Evaluations

All of the patients were divided into 3 groups based on BMI (kg/m^2^) (Group A: ≤ 24.9; Group B: 25–29.9; Group C: ≥ 30) within each disease category. According to World Health Organization (WHO) definitions, Group A was classified as being normal weight, Group B as overweight, and Group C as having grade I obesity, while grade II obesity (BMI ≥ 35 kg/m^2^) is rare in our country. Age, gender, and BMI were evaluated for each disease category. Moreover, surgical time was also assessed based on BMI classification and gender differences for LAM, THA and TKA.

### Statistical analysis

For statistical analysis, we used non-repeated measures of ANOVA and post hoc Bonferroni correction was applied to compare age and BMI among three groups. Categorical variable such as gender was compared using the chi-square test. The threshold for statistical significance was set at a *P* < 0.05. In terms of age and surgical times in THA, TKA and LAM, non-repeated measures of ANOVA and post hoc Bonferroni correction with *P* < 0.05 indicating a significant difference were used among BMI classification. Regarding the surgical time based on gender difference, unpaired t-test was applied. The threshold for statistical significance was also set at a *P* < 0.05. All statistical analyses were done using the Microsoft Excel Statistical Package, version 2019 (Social Survey Research Information, Tokyo).

## Results

Patient demographics in each group were presented in Table 1. A total of 269, 470, and 422 patients were allocated to the HOA category (THA), the KOA category (TKA), and the LCS category (LAM), respectively. The TKA category included the oldest patients and largest BMI, compared to the THA and the LAM categories. Regarding gender difference, LCS was more common in males than in females, while opposite phenomenon was observed in the HOA and the KOA categories. The number of patients based on BMI in LCS was 264 in Group A, 134 in Group B, and 24 in Group C. The number in HOA was 175 in Group A, 76 in Group B, and 18 in Group C. The number in KOA was 195 in Group A, 195 in Group B, and 80 in Group C.

**Table 1:** Patient demographics in each group (mean ± SD)

	THA	TKA	LAM	*P* Value^a^
Age (yrs)	66.4 ± 10.6	74.9 ± 8.2*	70.9 ± 4.0**†	*P* < 0.001
Gender (Female / Male)	222 / 47	377 / 93	119/303**†	*P* < 0.001
Body Mass Index (kg/m^2^)	23.6 ± 3.9	26.3 ± 4.4*	24.4 ± 3.5**†	*P* < 0.001
Surgical time (min)	107.2 ± 31.7	99.0 ± 20.1*	55.2 ± 22.7**†	*P* < 0.001

^a^ Values obtained using ANOVA

* *P* < 0.05 between THA and TKA

** *P* < 0.05 between TKA and LAM

† *P* < 0.05 between THA and LAM

**Fig. 2:**
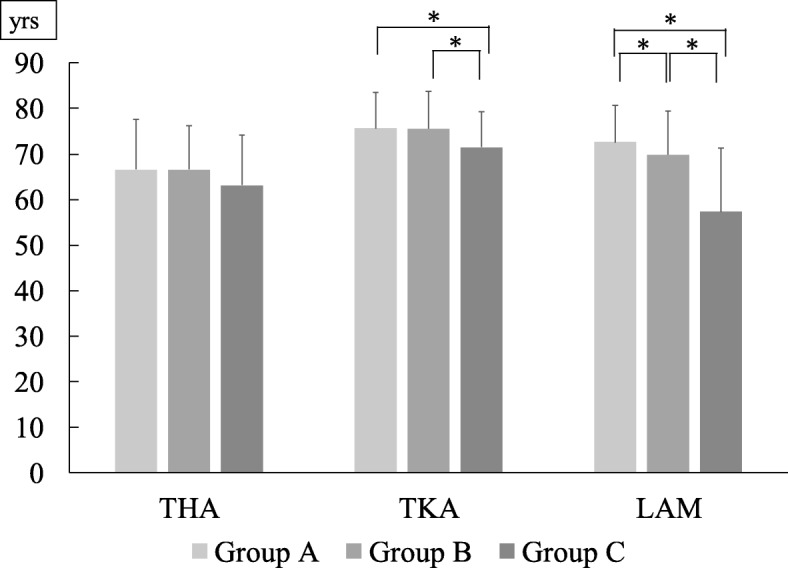
Mean age based on BMI for each disease category (* *P* < 0.05)

**Fig. 3:**
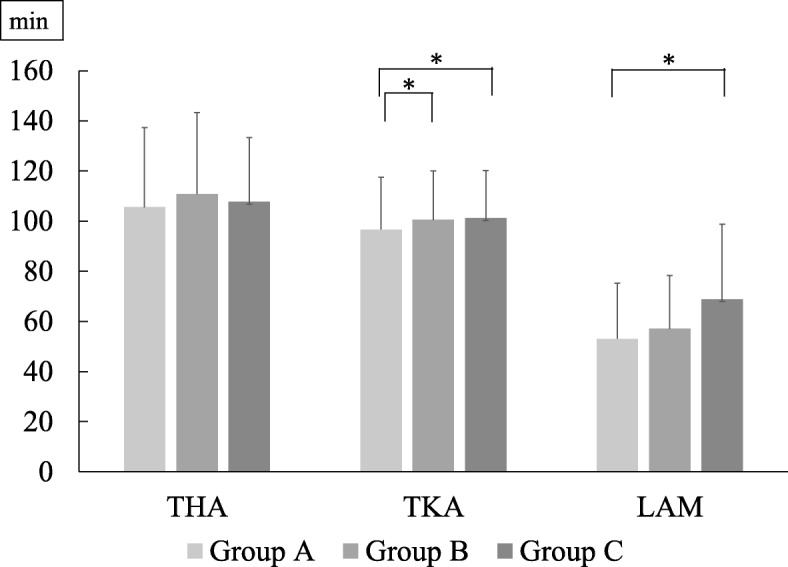
Mean surgical times based on BMI for each disease category (* *P* < 0.05)

**Fig. 4:**
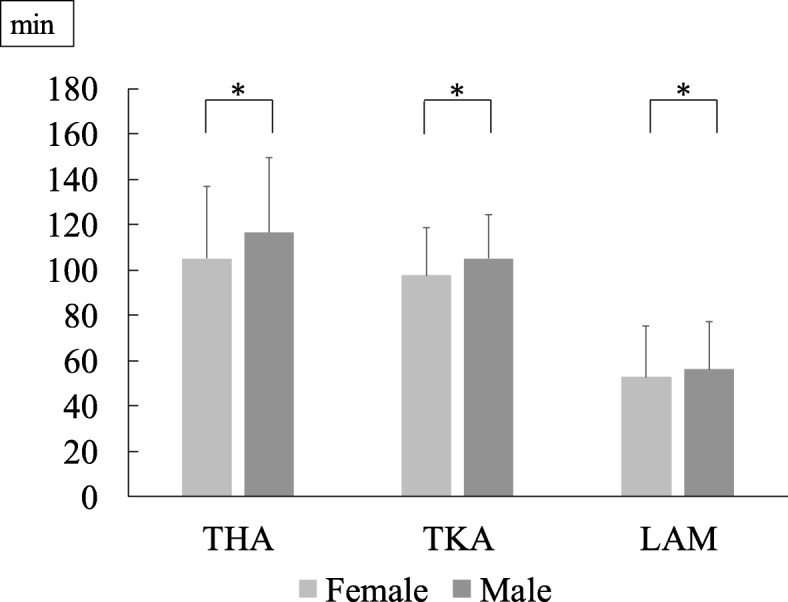
Mean surgical times based on gender difference for each disease category (* *P* < 0.05)

Mean ages based on BMI for each disease category are presented in Fig. 2. The heaviest group (Group C) was significantly younger than Groups A or B in the KOA category and the LCS category, while no significant difference was observed in the HOA category. Mean surgical times based on BMI was found in Fig. 3. Surgical time was significantly longer in patients with overweight or grade I obesity than in those with normal weight in TKA and LAM. Similarly, the time was not significantly different in THA. Mean surgical times based on gender difference was found in Fig. 4. For each surgery, surgical times were significantly longer in male patients than in female patients.

## Discussion

The present results partly supported the hypothesis that disease characteristics and surgical times would be strongly affected by BMI for three different categories. The main finding of the present study was that the heaviest group (Group C) was significantly younger than Groups A or B in the KOA category and the LCS category, and surgical time was significantly longer in patients with overweight and grade I obesity than in those with normal weight in TKA and LAM, while no significant difference was found in THA. Therefore, the HOA category (THA) was little affected by obesity in terms of disease characteristics and surgical time, compared to TKA and LAM.

Obese patients, who required surgical treatment, were significantly younger compared to those with normal body weight in the KOA and the LCS categories in the present study. According to previous studies, patient age < 65 years, male sex, BMI ≥30 kg/m^2^ were risk factors for operative time exceeding 120 min in total joint arthroplasties, which was associated with increased short-term morbidity and mortality after primary TKA and THA [16]. Concerning LCS, Burgstaller JM et al. suggested that obese patients could expect clinical improvement after lumbar decompression for LCS, but the percentage of patients with a meaningful improvement might be lower in the group of obese patients than in the group of patients with under-, normal, and pre-obese weight [2]. Because obesity is a modifiable risk factor for progression of degenerative changes, it is possible that younger patients may avoid surgical treatment especially for KOA and LCS, if they are able to maintain appropriate body weight. In the HOA category, there were no significantly differences of age based on BMI. Presumably, the reason was that secondary changes were more common in patients with HOA than in those with KOA in our country.

Regarding surgical time, the effect of BMI was more obvious in TKA and LAM than in THA. Namely, orthopedic surgeons should take caution and make an effort to reduce surgical time for overweight or obese patients with knee OA and LCS. Based on previous literatures, both TKA and THA would be affected by BMI. However, surgical time in THA was not significantly different in the present study among three BMI groups. Specifically, one of reasons were that grade II obesity (BMI ≥ 35 kg/m^2^) was rare in our country and BMI was lowest in the HOA category. On the other hand, Lozano LM et al. indicated that severely and morbidly obese patients (BMI > 35 kg/m^2^) undergoing TKA surgery should not require longer operative times or hospital stays than normal-weight or overweight patients [18]. As morbidly obese patients (BMI ≥ 35 kg/m^2^) were treated by a specific specialized surgical team in their study, the study was successful in reducing surgical times for obese patients. Concerning the gender difference, significantly longer surgical time was observed in male patients than in female patients in the present study for each surgery. Similarly, Kosashvili Y et al. suggested that TKA in men required more time than in women because of the complexity of exposure and achievement for the desired alignment of the components [19]. In addition to TKA, similar results were obtained for both THA and LAM in the present investigation.

Several limitations should be noted in the present study. First, the total numbers of patients were different among disease categories. Second, the present study was not based on an epidemiologic protocol, and was limited to surgical cases. Therefore, the diagnosis and surgical indication depended on each physician’s decision. Third, detailed information about disease severity was not included. Lastly, surgical details regarding skill of assistants in each surgery were unknown. However, since the current study is the first to compare the disease characteristics and surgical times of degenerative spinal deformity and osteoarthritis of the lower extremities based on BMI, the results of the present study offer useful information when considering the disease characteristics and surgical time of BMI-related degenerative diseases at the weight bearing joints including low back spine and lower limb.

## Conclusions

The KOA category and the LCS category were strongly affected by BMI, and surgical times in TKA and LAM were significantly longer for overweight or obese patients, while the HOA category and surgical time in THA were less affected by BMI. Therefore, the HOA category (THA) was little affected by obesity in terms of disease characteristics and surgical time, compared to TKA and LAM. Hence, orthopedic surgeons should take caution and make an effort to reduce surgical time for overweight or obese patients with knee OA and LCS.

## Data Availability

All supporting data can be provided based on request to MY or KH.

## Abbreviations

BMI: Body mass index
CSM: Cervical spondylotic myelopathy
HOA: Hip osteoarthritis
KOA: Knee osteoarthritis
LAM: Laminoplasty
LCS: Lumbar spinal canal stenosis
THA: Total hip arthroplasty
TKA: Total knee arthroplasty
